# Selinexor decreases HIF-1α via inhibition of CRM1 in human osteosarcoma and hepatoma cells associated with an increased radiosensitivity

**DOI:** 10.1007/s00432-021-03626-2

**Published:** 2021-04-15

**Authors:** Moritz von Fallois, Friederike Katharina Kosyna, Markus Mandl, Yosef Landesman, Jürgen Dunst, Reinhard Depping

**Affiliations:** 1 Universität Zu Lübeck, Institut Für Physiologie, Working Group Hypoxia, Ratzeburger Allee 160, 23562 Lübeck, Germany; 2 Karyopharm Therapeutics, 85 Wells Ave, Newton, MA 02459 USA; 3 Universitätsklinikum Schleswig-Holstein, Campus Kiel–Klinik für Strahlentherapie, Arnold-Heller-Straße 3, 24105 Kiel, Germany

**Keywords:** Hypoxia, Nuclear transport, Radiotherapy, HIF, CRM1, Selinexor

## Abstract

**Background:**

The nuclear pore complexes (NPCs) are built of about 30 different nucleoporins and act as key regulators of molecular traffic between the cytoplasm and the nucleus for sizeable proteins (> 40 kDa) which must enter the nucleus. Various nuclear transport receptors are involved in import and export processes of proteins through the nuclear pores. The most prominent nuclear export receptor is chromosome region maintenance 1 (CRM1), also known as exportin 1 (XPO1). One of its cargo proteins is the prolyl hydroxylase 2 (PHD2) which is involved in the initiation of the degradation of hypoxia-inducible factors (HIFs) under normoxia. HIFs are proteins that regulate the cellular adaptation under hypoxic conditions. They are involved in many aspects of cell viability and play an important role in the hypoxic microenvironment of cancer. In cancer, CRM1 is often overexpressed thus being a putative target for the development of new cancer therapies. The newly FDA-approved pharmaceutical Selinexor (KPT-330) selectively inhibits nuclear export via CRM1 and is currently tested in additional Phase-III clinical trials. In this study, we investigated the effect of CRM1 inhibition on the subcellular localization of HIF-1α and radiosensitivity.

**Methods:**

Human hepatoma cells Hep3B and human osteosarcoma cells U2OS were treated with Selinexor. Intranuclear concentration of HIF-1α protein was measured using immunoblot analysis. Furthermore, cells were irradiated with 2–8 Gy after treatment with Selinexor compared to untreated controls.

**Results:**

Selinexor significantly reduced the intranuclear level of HIF-1α protein in human hepatoma cells Hep3B and human osteosarcoma cells U2OS. Moreover, we demonstrated by clonogenic survival assays that Selinexor leads to dose-dependent radiosensitization in Hep3B-hepatoma and U2OS-osteosarcoma cells.

**Conclusion:**

Targeting the HIF pathway by Selinexor might be an attractive tool to overcome hypoxia-induced radioresistance.

## Introduction

To cure cancer is a major problem in the world and new questions arise faster than old ones are solved. The disease is characterized by different genetic aberrations leading to uncontrolled cell division and distraction of the surrounding tissue (Hanahan and Weinberg 2011). Therapy strategies include surgical and medical treatment, radiation therapy or a combination with one of these approaches.

All cellular proteins are embedded in their specific microenvironment. Hence, the functions of proteins are manifold: they serve as part of the cytoskeleton, catalyze biochemical reactions and are part of important signaling cascades acting for example as transcription factors.

Eukaryotic cells are structural and functional compartmentalized resulting in the spatial separation of cytoplasm and nucleus. These two compartments are separated by the nuclear envelope (D'Angelo and Hetzer 2008). In the nucleus, DNA is transcribed into mRNA, which is translated into proteins via ribosomes in the cytosol. Therefore, mRNA and translated proteins have to shuttle between the nucleus and the cytoplasm through nuclear pore complexes (NPCs). These NPCs are built of about 30 different nucleoporins (Beck and Hurt 2017). Smaller proteins can passively diffuse through the NPCs; whereas, macromolecules greater than 40 kDa need facilitated transport (Stelma et al. 2016).

The translocation is mediated by the protein superfamily of karyopherins, which function as mobile transport receptors. Simplified, they can be divided in import and export receptors and recognize special localization signals, the nuclear localization signal (NLS) and the nuclear export signal (NES), respectively (Turner and Sullivan 2008). The best characterized nuclear export receptor is the export protein chromosome region maintenance 1 (CRM1), also known as exportin1 (XPO1) (Ishizawa et al. 2015). In the nucleus, CRM1 forms a complex with the cargo protein and Ras-related nuclear guanosine triphosphate (RanGTP) followed by export into the cytosol. Here, RanGTP is hydrolyzed into RanGDP and the trimer dissociates (Dickmanns et al. 2015).

CRM1 has been shown to be involved in the export of over 200 proteins including the tumor suppressor protein p53 and the protein family of prolyl hydroxylases (PHDs) (Xu et al. 2012; Hutten and Kehlenbach 2007). PHDs belong to a family of proteins regulating the stability of other proteins via hydroxylation in an oxygen-dependent manner. The most abundant member of this family is PHD2, the key regulator of hypoxia-inducible factor (HIF-1). HIFs play a major role in the transcriptional response to hypoxia. They promote angiogenesis by inducing the expression of transcriptional factors including vascular endothelial growth factor (VEGF) (Du et al. 2008). HIF-1 consists of HIF-1β, which is constitutively expressed and HIF-1α, which is degraded in normoxia; whereas, it is stabilized in hypoxia. Several studies demonstrate that HIF-silencing induces anti-tumor effects under hypoxic conditions (Choi et al. 2014). In addition, it has been shown that stabilization of HIFs in hypoxia decreases radiosensitivity; whereas, a HIF knockdown increases radiosensitivity (Strofer et al. 2011).

In many cancer cells, CRM1 is upregulated and its overexpression is directly linked to cancer cell survival (Watt et al. 2009). In line with this, nuclear protein levels of CRM1-cargo proteins such as p53 and PHDs are decreased in some types of cancer. Thus, the inhibition of transport proteins such as CRM1 seems to be an interesting way in understanding cell function on the one hand and establishing new therapy concepts on the other hand (Kosyna 2018).

The new pharmaceutical Selinexor is a selective inhibitor of nuclear export (SINE). Selinexor was designed using consensus-induced fit docking (cFID), a method which allows to simulate the interaction between different molecules on the computer. Structure analysis demonstrate that SINE bind specific to the cysteine-residue of CRM1 which binds to the NES of cargo proteins. It is not hydrolysated by CRM1 and although it is a covalent binding, it is not an irreversible but rather a slow-reversible inhibitor (Fung and Chook 2014). Selinexor is currently tested in three different Phase-3 clinical trials in solid and hematological tumors (Kauffman 2020). Furthermore, it was approved for refractory multiple myeloma by the FDA in 2019 (Dolgin 2019). Selinexor inhibits CRM1 via reversible covalent binding to cysteine 528 in the cargo binding region of CRM1, leading to a higher nuclear concentration of proteins translocated by CRM1 (Gounder et al. 2016).

In this study, we focused on the effects of Selinexor on the HIF-pathway. We demonstrate that Selinexor decreases nuclear HIF-1α protein levels in human hepatoma Hep3B cells and human osteosarcoma U2OS cells under hypoxic conditions. To the best of our knowledge, we show for the first time, that Selinexor treatment increases radiosensitivity in both cell lines. Thus, we further underline the potential of Selinexor in cancer treatment.

## Results

### U2OS and Hep3B cells are susceptible to Selinexor treatment under normoxic and hypoxic conditions

**Fig. 1:**
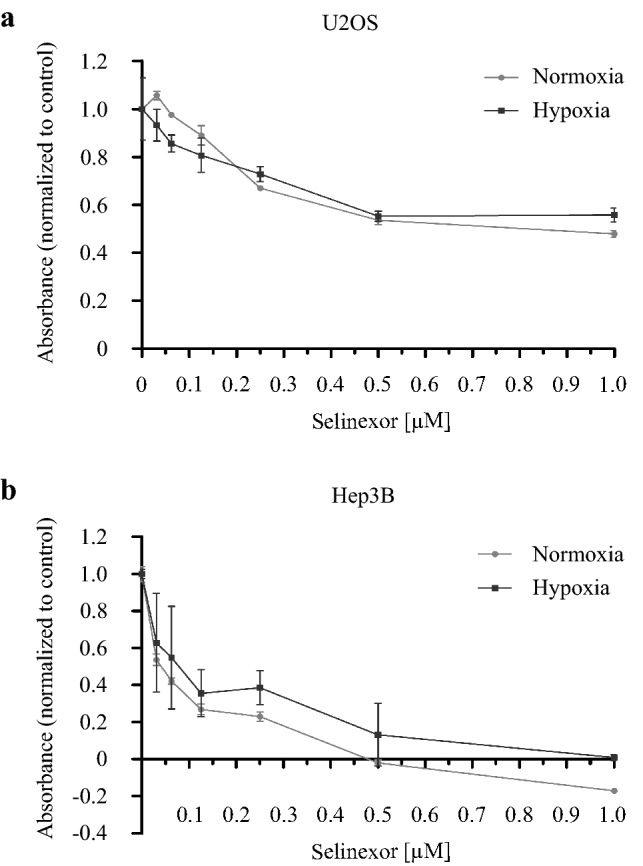
Effect of Selinexor on cell viability. **a** U2OS and **b** Hep3B cells were treated with concentrations of Selinexor from 0.031 to 1.000 µM (0.031 µM, 0.063 µM, 0.125 µM, 0.250 µM, 0.500 µM, 1.000 µM) for 72 h under normoxic and hypoxic conditions. Subsequently, cell viability was assayed using the alamarBlue™ Reagent (Invitrogen) according to the manufacturer´s protocol. Values are presented as mean ± SD (*n* = 5)

Tumor hypoxia causes a more malignant phenotype of solid tumors and contributes to therapy resistance. To investigate whether hypoxia might promote the resistance of U2OS and Hep3B cells to Selinexor, dose–response curves were conducted under normoxic and hypoxic conditions. As expected, viability of U2OS and Hep3B cells decreased in a dose-dependent manner when incubated with Selinexor in doses of 0.031 µM up to 0.5 µM for 72 h in normoxia or hypoxia. Higher concentrations than 0.5 µM revealed no significant decrease in cell viability (Fig. 1). In addition, no statistically significant differences were observed under hypoxic conditions. These results indicate that both cell models respond to CRM1 inhibition even under oxygen deprivation. It should be noted, that the slight increase in U2OS cell viability in normoxia after treatment with 0.3 µM Selinexor is not statistically significant.

### Selective inhibition of CRM1 affects the nuclear concentration of HIF-1α and p53

**Fig. 2:**
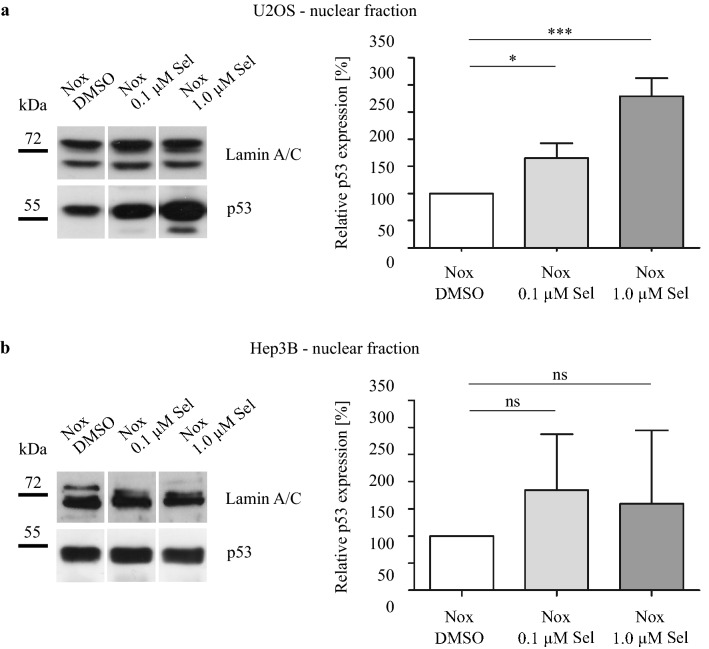
Immunoblot analysis of intranuclear p53 protein level. a) U2OS and b) Hep3B cells were treated with DMSO, 0.1 µM Selinexor or 1.0 µM Selinexor under normoxia for 24 h and fractionated. Hep3B cells are deficient for the tumor suppressor protein p53 and were transiently transfected with a p53-pcDNA-plasmid. Relative protein levels of intranuclear p53 protein in % ± SD compared to DMSO control are shown. One-way ANOVA with Tukey posttest with *p < 0.05 and ***p < 0.001 (*n* = 3)

To analyze whether CRM1 was blocked by Selinexor, we determined the intranuclear level of p53 as control. Human hepatoma Hep3B cells are inherently deficient for p53, hence they were transiently transfected with a p53-pcDNA-plasmid. As expected, Selinexor treatment increased intranuclear p53 protein level in U2OS cells. In Hep3B cells, we observed a trend towards an increase in intranuclear p53 protein level, which was not statistically significant (Fig. 2). These results hint toward the susceptibility of U2OS and Hep3B cells to CRM1 inhibition.

**Fig. 3:**
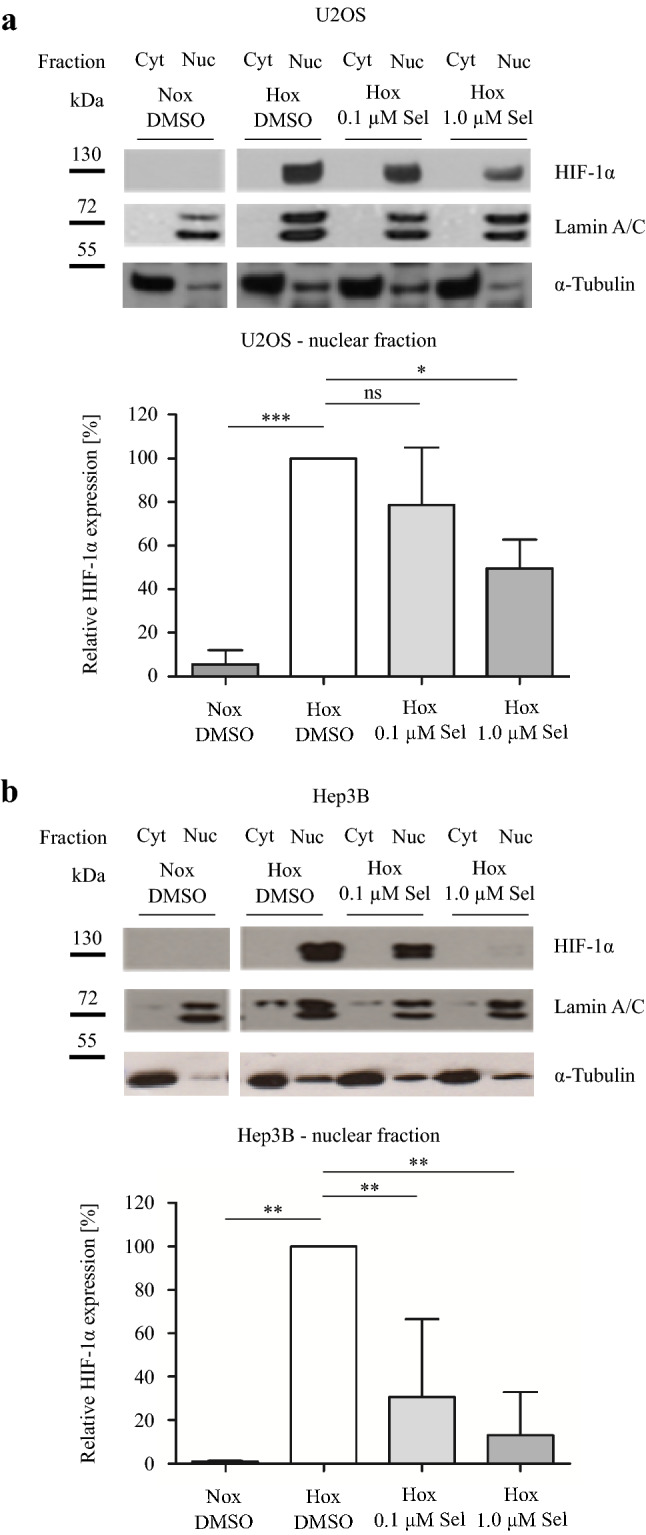
Immunoblot analysis of intranuclear HIF-1α protein levels **a** U2OS and **b** Hep3B cells were treated with DMSO, 0.1 µM Selinexor or 1.0 µM Selinexor under normoxia or hypoxia for 24 h and fractionated. Representative immunoblot images of *n* = 3 independent experiments are shown. Specific signals were quantified densitometrically and normalized to hypoxic DMSO-treated control cells. Values are presented as mean ± SD with *p < 0.05, **p < 0.01 and ***p < 0.001 (*n* = 3)

Next, we aimed to investigate the effect of Selinexor on the subcellular localization of HIF-1α. Therefore, U2OS and Hep3B cells were treated with 0.1 or 1.0 µM Selinexor for 24 h under normoxic and hypoxic conditions. The nuclear concentration of HIF-1α was measured by immunoblot analysis after separation of cells in cytoplasmic and nuclear fractions. Integrity of nuclear and cytoplasmic protein fractionation was checked by immunoblot analysis of nuclear markers lamin A/C and cytoplasmic marker α-tubulin (Fig. 3). Nuclear and cytoplasmic extracts have minimal contamination between fractions which represents sufficient purity. Faint bands of α-tubulin in nuclear extracts can be explained by the presence of α-tubulin at the cytoplasmic site of the nuclear membrane, while minimal levels of lamin A/C in cytoplasmic extracts may represent minimal levels of lamin cleavage products.

As shown in Fig. 3, inhibition of CRM1 by Selinexor decreased the nuclear protein level of HIF-1α in a dose-dependent manner. In U2OS cells, we demonstrated a decrease of approximately 50% and in Hep3B even a drop of more than 80% after treatment with 1.0 µM Selinexor under hypoxic conditions. These findings indicate that the function of CRM1 is critical in the regulation of nuclear transport processes in the HIF-signaling pathway.

### Inhibition of CRM1 decreases radioresistance of osteosarcoma and hepatoma cells

Stroefer et al*.* could show that knockdown of HIF-1α leads to an increased response on radiotherapy in human cancer cell models (Strofer et al. 2011). Following this, we supposed that the inhibition of CRM1 by Selinexor in U2OS and Hep3B cells could lead to an increased radiation response due to decreased HIF-1α protein levels. Therefore, cells treated with Selinexor for 24 h were irradiated with 2, 4, 6 or 8 Gy and clonogenic survival assays were performed.

**Fig. 4:**
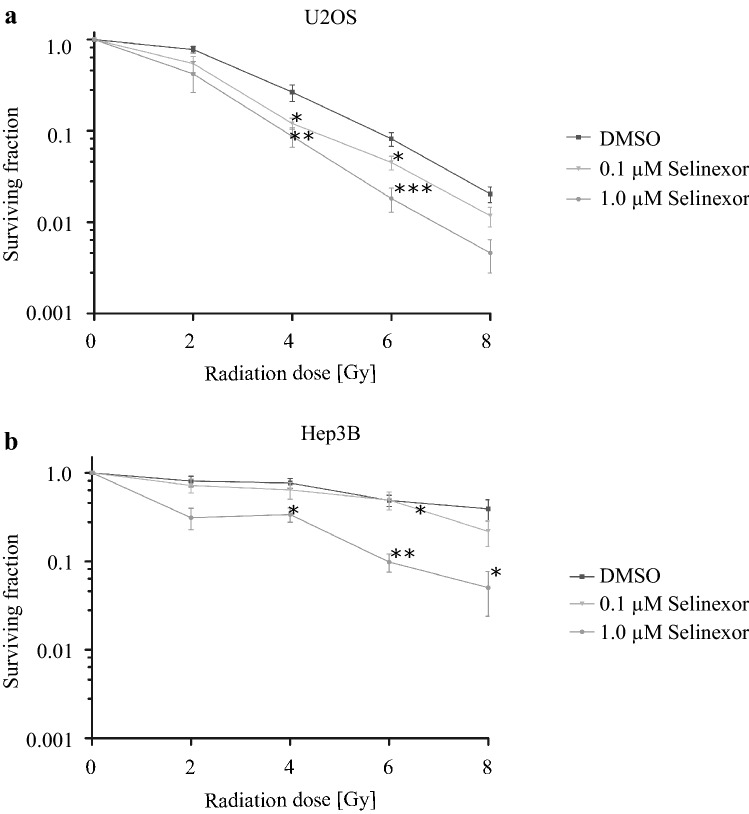
Irradiation of Selinexor-treated tumor cells. **a** U2OS and **b** Hep3B cells were incubated with Selinexor or DMSO followed by X-ray irradiation and incubation for further 9 days. Afterwards, colonies were stained with crystal violet and counted and compared to untreated control cells. Values are presented as mean ± SD with *p < 0.05, **p < 0.01 and ***p < 0.001 (*n* = 6)

Survival fractions are presented in Fig. 4 in percent of irradiated and Selinexor-treated cells compared to untreated control cells. We demonstrate a statistically significant reduction in the radioresistance in both cell lines after treatment with Selinexor.

## Discussion

Among others, the HIF-signaling pathway seems to be important especially in dysplastic tissues. Due to its growth and a consequently deficient blood flow, dysplastic tissue often develops hypoxic regions. Following this, the HIF-signaling pathway is activated leading to multiple effects such as an increasing neovascularization or an increased invasiveness and radioresistance in these tumors (Baumann et al. 2016; Terry et al. 2018).

Although classic chemotherapies have been an indispensable component of anti-cancer therapies, new target-directed therapies become more and more part of daily medical practice. The idea is simple: in a targeted manner, these new pharmaceuticals can inhibit the biomolecular reasons for cancer-dependent up- or downregulated enzymes, which lead to tumorigenesis.

One of these promising targets is the NPC, a large multimeric protein complex of 125 MDa consisting out of approximately 30 different nucleoporins (Pickens and Tripp 2018). Its function is critical for the subcellular localization of enzymes and other macromolecules that are involved in the development of cancer (Fukuda et al. 1997). Attempts are made to inhibit parts of the nuclear export mechanisms that are involved in the intracellular transport of drug targets and tumor suppressors (El-Tanani et al. 2016). In its highly conserved NES binding site, CRM1 contains a cysteine residue on position 528 which is critical for the interaction between CRM1 and the NES of a specific cargo (Kosyna 2018). For a variety of solid tumors, overexpression of CRM1 has been shown. In patients with osteosarcoma and in preclinical models of hepatocellular carcinoma cell lines, a correlation between the expression of CRM1 and cancer proliferation was demonstrated (Yao et al. 2009; Zheng et al. 2014).

In this context the intracellular transport of proteins belonging to the family of prolyl hydroxylases is of particular interest (Depping et al. 2015). PHDs are involved in the degradation process of HIFs. Especially for PHD2, it has been shown that the hydroxylation of HIF-1α can take place in the cytoplasm as well as in the nucleus; whereas, the activity of PHD2 is higher in the latter one (Berchner-Pfannschmidt et al. 2008). Reasonably, it might be assumed, that the inhibition of CRM1 may lead to accumulation of PHD2 in the nucleus followed by an enhanced degradation of HIF-1α.

Over the last decades, attempts have been made to identify inhibitors of nuclear export. Typically, research on nuclear export inhibitors has concentrated on direct inhibition of CRM1 leading to the development of SINE. The first promising pharmaceutical out of this group with effects on CRM1 was Leptomycine B (LMB), which was discovered in the early eighties in Streptomyces *spp* and was established as a new antifungal pharmaceutical. LMB alkylates a reactive cysteine residue (cysteine 528) on CRM1 in a covalent and irreversible manner inhibiting CRM1-binding to the NES of a cargo protein. However, in a phase I clinical trial LMB was found to have severe toxicities and therefore its clinical use was discarded (Newlands et al. 1996). Other inhibitors such as the aromatic ketone Trans-chalcone (Silva et al. 2018) and CBS9106 (Sakakibara et al. 2011) were developed, but none of these candidates reached clinical practice so far.

A promising new candidate in the family of SINEs is Selinexor, which uses the same target as LMB. It binds specifically and reversibly (unlike LMB) to the cysteine residue 528 on CRM1 (Neggers et al. 2015). Up to now, Selinexor has been tested in many kinds of cancer being quite well-tolerated in phase I and II clinical trials. In different studies, it has been combined with chemotherapeutics and targeted therapies such as platinum or tyrosin kinase inhibitors in vitro and in vivo with mainly promising results (Corno et al. 2018; Nie et al. 2018). By now, two research groups combined Selinexor with radiation in a preclinical model of rectal cancer and an in vitro and in vivo study of glioblastoma cells and found promising effects (Ferreiro-Neira et al. 2016; Wahba et al. 2018).

In line with these reports, we could recently show that Selinexor inhibits transcriptional-HIF activity, HIF-target gene expression and 3D spheroid growth of cancer cells (Depping et al. 2019). In this follow-up study, we could demonstrate that the inhibition of CRM1 by Selinexor decreases the intranuclear HIF-1α protein level in human osteosarcoma and human hepatocellular carcinoma cells accompanied by an enhanced radiation response in both cell lines. Since it has been shown that PHD2 is a CRM1 cargo protein and the hydroxylase activity of PHD2 strongly depends on its subcellular localization, it can be assumed that PHD2 accumulates in the nucleus after CRM1 inhibition (Steinhoff et al. 2009; Pientka et al. 2012). By this, decreased nuclear HIF-1α protein levels after Selinexor treatment may be explained.

Ströfer et al*.* showed, that there is a link between the degradation or knockout of HIF-1α and the viability and radiosensitivity of cancer cells in vitro (Strofer et al. 2011). In addition, Mucha-Malecka et al*.* showed in a long-term retrospective study in cancer patients, that low levels of hemoglobin are associated with poor prognosis (Mucha-Malecka et al. 2019). The underlying causes are manifold (Tang et al. 2018), but as we know, that lower hemoglobin levels lead to relative hypoxia in peripheral tissue, we can assume that this leads to higher HIF-1α levels and potential tumorigenesis.

Apart from the effect of Selinexor on radiosensitivity of cancer cells mediated by the HIF-signaling pathway several studies showed the potential of Selinexor to diminish DNA repair after damage. For example, Ranganathan et al*.* could show that Selinexor combined with Topoisomerase II inhibitors leads to a prolonged survival in the AML mouse model (Ranganathan et al. 2016).

The findings of this study clearly demonstrate that the CRM1 inhibition leads to an increased radiation response. This can be explained by different molecular mechanisms discussed above. In future studies, the importance of the intracellular localization for the regulation of different signaling pathways should be investigated with special emphasis on HIF-1α depleted cells. Moreover, the significance of inhibition of DNA damage repair in cells treated with Selinexor has to be elucidated to understand the underlying mechanisms on irradiated cells.

## Materials and methods

### Antibodies, chemicals and cell culture

Antibodies used were anti-HIF-1α (1:1000, R&D Systems, Minneapolis, MN, USA), anti-p53 (1:1000, Abcam, Cambridge, UK), anti-Lamin A/C (1:1000) and anti-α-Tubulin (1:5000, both Santa Cruz Biochtechnology, Dallas, TX, USA).

Cell lines were purchased by the German Collection of Microorganisms and Cell Cultures GmbH (DMSZ, Braunschweig, GER). They were routinely tested for mycoplasma contamination with a PCR-based assay.

The human cell lines U2OS (human osteosarcoma, doubling time 50–60 h) and Hep3B (human hepatoma, doubling time 40–50 h) were grown in DMEM and RPMI-1640 culture medium (Gibco, Darmstadt, GER), respectively, containing 10% fetal calf serum (FCS, Gibco, Grand Island, NY, USA) and 100 IU/ml penicillin/100 µg/ml streptomycin (PAA Laboratories, Pasching, AUT) at 37 °C in a humidified 5% CO_2_ incubator. To study hypoxic conditions, cells were placed in a humidified atmosphere containing 3% O_2_, 5% CO_2_ and balanced N_2_ (Heracell Vios 160i Co2-Incubator, Thermo scientific, Waltham, MA, USA).

Selinexor (KPT-330) was kindly provided by Karyopharm Therapeutics (Newton, MA, USA). Selinexor was dissolved in dimethyl sulfoxide (DMSO, Sigma Aldrich, St. Louis, MO, USA) to a concentration of 10 mmol.

### Transient transfection

Hep3B cells are deficient for the tumor suppressor protein p53. To use p53 as control for Selinexor treatment, Hep3B cells were transiently transfected with p53–pcDNA–plasmid using Genejuice transfection reagent (Merck Millipore, Burlington, MA, USA). Cells were grown to 80% confluency on 10 cm Petri dishes before transfection according to the manufacturer’s protocol.

### Cell viability assay

U2OS and Hep3B cells were seeded in 96-well plates at a density of 1 × 10^4^ cells per well. The next day, cells were treated with concentrations of Selinexor from 0.031 to 1.000 µM (0.031 µM, 0.063 µM, 0.125 µM, 0.250 µM, 0.500 µM, 1.000 µM) and subjected to hypoxic or normoxic conditions for 72 h. Each condition was assayed in four technical replicates. Subsequently, cell viability was determined using the alamarBlue™ Cell Viability Reagent (Invitrogen, Darmstadt, GE) according to the manufacturer’s protocol (590 nm, Mithras LB 940, Berthold Technologies GmbH & Co. KG, Bad Wildbad, Germany).

### Immunoblot analysis

Following transfection and treatment with Selinexor for 24 h, cells were incubated under normoxic or hypoxic (3% O_2_) conditions for 24 h. Cells were washed with ice-cold PBS and separated into nuclear and cytoplasmic fractions using the NE-PER Nuclear and Cytoplasmic extraction reagents purchased by Thermo Fisher (Waltham, MA, USA) according to the manufacturer’s protocol.

Protein concentration was determined using Bio-Rad DC Protein Assay (Bio-Rad, Hercules, CA, USA). Proteins were separated by SDS-Page and transferred onto polyvinylidene difluoride membrane (PVDF, Merck Millipore, Burlington, MA, USA) by semi-dry electroblotting. The blots were incubated with primary antibodies for 24 h and with HRP-conjugated secondary antibodies (Dako Denmark, Glostrup, DNK) in a concentration of 1:1000 up to 1:5000for 1 h and finally detected by electrochemiluminescence (ECL, Bio-Rad, Hercules, CA, USA).

### Clonogenic survival assay

For clonogenic assays, cells were seeded onto 24-well plates, incubated overnight and treated with Selinexor in a concentration of 0.1 or 1.0 µM for 24 h. After irradiation with doses of up to 8 Gy (Varian Clinac DHX 5024, Palo Alto, CA, USA), cells were harvested using Accutase (Thermo Fisher, Waltham, MA, USA) and counted using trypan-blue staining and measurement using the Cellometer™ Auto T4 (Nexcelcom Bioscience LTD, Lawrence, MA, USA). 400–800 living cells were seeded in 6-well culture dishes in triplicates. After incubation for 9 days, colonies were fixed with crystal violet and dried. Colonies out of six independent experiments were counted randomized and blind and the clonogenic survival was calculated in comparison to untreated control cells.

### Statistics

Statistical analysis was performed using GraphPad Prism Software (GraphPad Software, La Jolla, CA, USA). To compare two treatment groups the One-way ANOVA with Tukey post hoc test was performed. Graphs are shown as mean ± standard deviation (SD). Immunoblot experiments were performed three times (*n* = 3) and experiments of clonogenic survival at least six times (*n* = 6).

## Abbreviations

CRM1: Chromosome region maintenance 1
HIF-1α: Hypoxia-inducible factor-1α
NES: Nuclear export signal
NLS: Nuclear localization signal
NPC: Nuclear pore complex
PHD2: Prolyl hydroxylase 2
RanGTP/GDP: Ras-related nuclear guanosine triphosphate/diphosphate
SINE: Selective inhibitor of nuclear export
VEGF: Vascular endothelial growth factor
XPO1: Exportin 1
